# Role of KNDy neurons in puberty onset in male offspring following prenatal androgen exposure

**DOI:** 10.1530/EC-25-0209

**Published:** 2025-07-17

**Authors:** Runfei Ge, Yun Zhang, Yongting Yuan, Tingting Li, GuiYu Qiu, Shuaijun Guo, Lianguo Fu

**Affiliations:** ^1^ Department of Child and Adolescent Health, School of Public Health, Bengbu Medical University, Bengbu, China; ^2^ Centre for Community Child Health, Murdoch Children’s Research Institute, Melbourne, Australia; ^3^ Department of Pediatrics, University of Melbourne, Melbourne, Australia

**Keywords:** prenatal, testosterone, offspring, puberty onset, KNDy neurons

## Abstract

**Graphical abstract:**

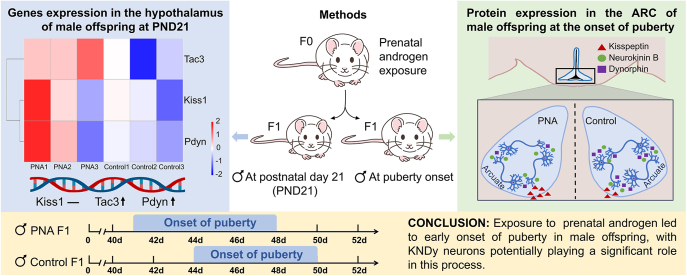

**Abstract:**

**Objective**

Emerging evidence links prenatal androgen excess to altered pubertal timing, yet the neuroendocrine mechanisms mediating this effect in male offspring remain poorly characterized. This study aimed to investigate the effects of prenatal androgen exposure on the timing of puberty onset in male offspring and the role of KNDy neurons in this process.

**Methods**

Eight-week-old pregnant Sprague–Dawley rats (*n* = 16) were randomized into control (olive oil) and prenatal androgen (PNA, testosterone injection) groups (*n* = 8 per group). Hypothalamic samples of six male offspring rats at postnatal day (PND) 21 (*n* = 3 per group) were collected for transcriptome analysis. The time of puberty onset was recorded in 36 male offspring (*n* = 18 per group). Serum samples and hypothalamic tissue from 12 male offspring rats (*n* = 6 per group) were collected, and GnRH, LH, FSH, and kisspeptin 1 protein levels were measured by ELISA. The mRNA levels of *Kiss1*, *Tac3*, and *Pdyn* were measured by real-time qPCR. In the brains of six male offspring rats (*n* = 3 per group), protein levels of kisspeptin, neurokinin B (NKB), and dynorphin (Dyn) in the arcuate nucleus (ARC) were measured using immunohistochemistry.

**Results**

Compared to controls, PNA male offspring rats showed significantly earlier puberty onset (*P* < 0.001). At PND21, *Tac3* (*P* < 0.01) and *Pdyn* (*P* < 0.05) expression levels increased significantly in PNA male offspring rats. At puberty, ARC kisspeptin protein levels increased (*P* < 0.01), while dynorphin protein levels decreased (*P* < 0.01) in PNA male offspring rats.

**Conclusion**

Prenatal androgen exposure accelerates puberty onset in male offspring rats, likely by activating *Tac3* expression in early life and reducing ARC dynorphin inhibition of kisspeptin neurons at puberty.

## Introduction

Puberty is a critical phase of physical and psychological development during which adolescents experience the emergence of secondary sexual characteristics, sexual maturation, and personality formation. With socioeconomic development, the trend toward earlier puberty onset has become increasingly apparent (1). Studies have shown that the age of pubertal onset in girls, based on breast development, has advanced by nearly 3 months per decade (2). Similarly, data from Europe indicate a trend of earlier puberty onset in boys, assessed by testicular volume and genital staging (3). However, the early onset of puberty has been associated with higher risks of developing type 2 diabetes, cardiovascular diseases, obesity, and psychological conditions such as depression (4, 5, 6, 7), thus becoming a significant public health concern.

The onset of puberty is a complex process influenced by genetics, environment, nutrition, and hormones (8, 9, 10, 11). It involves the reactivation of the hypothalamic–pituitary–gonadal (HPG) axis. This axis is briefly active at birth, becomes suppressed during childhood, and is reactivated at the onset of puberty (12). Upon activation of GnRH neurons, gonadotropin-releasing hormone (GnRH) is released in pulses into the pituitary portal system and transported to the pituitary gland, stimulating the synthesis and release of gonadotropins (13). Recent studies have shown that hypothalamic GnRH secretion is regulated by various factors, including neuropeptide Y, pro-opiomelanocortin, gamma-aminobutyric acid neurons, and glutamate (14, 15, 16). However, the precise mechanisms by which HPG axis activation triggers puberty onset remain unclear. In recent years, KNDy neurons (kisspeptin/neurokinin B (NKB)/dynorphin (Dyn)-expressing neurons), which co-express kisspeptin, NKB, and Dyn in the arcuate nucleus (ARC), have been identified as key players in this process (17).

The co-expression of KNDy peptides in the ARC has been confirmed in various mammals, including sheep, mice, and rats (18, 19, 20). Previous studies have demonstrated that KNDy neurons act as the primary regulators of GnRH pulses (21, 22). KNDy neurons release NKB (encoded by *Tac3*), which recruits other KNDy neurons via the NKB receptor (NK3R) to secrete kisspeptin around GnRH neuron dendrites. Kisspeptin binds to its receptor, G protein-coupled receptor 54 (GPR54), activating GnRH neurons. Subsequently, KNDy neurons release dynorphin (encoded by *Pdyn*), which binds to kappa-opioid receptors (KOR) to terminate GnRH pulses. Animal studies have demonstrated that transfecting the *Kiss1* gene into the KNDy neurons of infertile female rats with *Kiss1* gene knockout can restore normal pulsatile gonadotropin release and folliculogenesis (23). In addition, *Kiss1* mRNA levels in the monkey hypothalamus increased with puberty (24). Administration of NK3R antagonists has been shown to modestly delay vaginal opening and reduce luteinizing hormone (LH) levels (25). These experimental findings suggest that KNDy neurons may play a critical role in regulating puberty.

Polycystic ovary syndrome (PCOS), a complex endocrine disorder characterized in part by hyperandrogenism, affects 5–20% of women of reproductive age worldwide (26). With the rising prevalence of PCOS, elevated androgen levels among women of childbearing age have become more common (27). Progress has been made in the study of the mechanisms by which androgen levels during pregnancy affect the offspring. When fetuses are exposed to high levels of androgens *in utero*, they face an increased risk of perinatal mortality, more congenital anomalies, and metabolic disorders (28). Prenatal androgen levels are associated with the development of girls (29, 30). In animal experiments, existing research has found that androgens during pregnancy advance the onset of puberty in male offspring by affecting the leptin–NPY pathway (31). Although epidemiological evidence indicates that excessive androgen exposure during pregnancy may disrupt the timing of puberty onset in offspring, the underlying neuroendocrine mechanisms remain incompletely understood. Notably, the role of hypothalamic KNDy neurons in this process represents a knowledge gap in the field. In the present study, we aimed to explore whether androgen exposure during pregnancy affects pubertal development by influencing the expression of KNDy neurons in the offspring and thus the role and mechanism of pubertal development. Therefore, we established a prenatal androgen (PNA) exposure rat model to determine the role of these neurons in the onset of puberty in offspring rats. This study provides scientific evidence for understanding the relationship between elevated prenatal androgen levels and pubertal development in male offspring.

## Materials and methods

### Animals and prenatally androgenized

Eight-week-old female and male Sprague–Dawley rats were purchased from Jinan Pengyue Laboratory Animal Breeding Co. and housed under controlled conditions with a 12 h light/12 h darkness cycle and a constant temperature of 25 ± 2°C. The rats had access to food and water *ad libitum*. After a 1 week acclimatization period, females and males were mated overnight. Female rats were monitored daily for the presence of post-copulatory plugs, and the day a plug was observed was designated as gestational day 1. Pregnant rats were randomly assigned to one of two groups: control or prenatal androgen (PNA), with eight rats in each group. On gestational days 15, 17, 19, and 21, the PNA group received subcutaneous injections of 2 mL of a 2.5 mg/mL testosterone solution at the nape of the neck (31, 32, 33). The control group received equivalent volumes of olive oil following the same schedule and procedure.

### Determination of onset of puberty and samples collection

Three male offspring at postnatal day 21 (PND21) from each of the control and PNA groups were randomly selected and anesthetized with 2% sodium pentobarbital (3 mL/kg), their hypothalamus was rapidly removed on ice, immediately placed in liquid nitrogen, and stored at −80°C for transcriptome sequencing and bioinformatics analysis. Preputial separation was used as an external marker of puberty onset in male offspring rats. Starting from PND21, 36 male offspring rats (18 in the control group and 18 in the PNA group) were monitored daily for signs of puberty onset. On the day of puberty onset, 12 male offspring rats (control:PNA = 6:6) were anesthetized as described above, and blood samples were collected, centrifuged, and stored at −80°C for subsequent analysis; hypothalamic samples were collected for real-time qPCR analysis. While six male offspring rats were anesthetized as described above, and the brains were fixed overnight in 4% formaldehyde for immunohistochemical evaluation.

### RNA sequencing

Total RNA was extracted from hypothalamic tissues of male offspring in both the control and PNA groups at PND21 using TRIzol reagent (Ambion/Invitrogen, USA). RNA integrity and quantity were assessed. mRNA with poly(A) tails was enriched, fragmented in Fragmentation Buffer, and reverse transcribed into cDNA. The resulting cDNA fragments were purified using the AMPure XP system (Beckman Coulter, USA) to select fragments between 370 and 420 bp in length. PCR amplification was performed using Phusion High-Fidelity DNA Polymerase, Universal PCR Primers, and Index (X) Primers. The PCR products were purified, and library quality was assessed using the Agilent Bioanalyzer 2100 system. Sequencing was carried out on the Illumina NovaSeq 6000 platform (Illumina, USA). Fluorescence signals captured by the sequencer were converted into sequencing reads, and the fragment sequences were obtained through computer analysis.

### Bioinformatics analysis

To ensure data quality and reliability, clean reads were obtained by removing reads containing adapters, poly-N, and low-quality bases from the raw data. The Q20, Q30, and GC content of the clean data were calculated, and all subsequent analyses were conducted using this high-quality dataset. The reference genome and gene model annotation files were downloaded from a genome database, and the reference genome index was constructed using Hisat2 v2.0.5. Paired-end clean reads were aligned to the reference genome using Hisat2 v2.0.5, and gene read counts were quantified using featureCounts v1.5.0-p3. Differential expression analysis between groups was performed using the DESeq2 R package (v1.20.0), with genes showing a *P* < 0.05 considered differentially expressed. The Benjamini–Hochberg method was applied to adjust *P*-values and control the false discovery rate. Gene ontology (GO) enrichment analysis and Kyoto Encyclopedia of Genes and Genomes (KEGG) pathway enrichment analysis of differentially expressed genes (DEGs) were conducted using the clusterProfiler R package, with GO or KEGG terms having an adjusted *P*-value (*Padj*) < 0.05 considered significantly enriched. Sequencing and bioinformatics analyses were carried out by Novogene Bioinformatics Technology Co. Ltd (China).

### Hormone assay

Enzyme-linked immunosorbent assay (ELISA) kits were used to quantify serum concentrations of GnRH (CSB-E08037r; Cusabio, China), LH (CSB-E12654r; Cusabio, China), FSH (CSB-E06869r; Cusabio, China), and kisspeptin 1 (CSB-E13434r; Cusabio, China). The detection ranges were 2–150 pg/mL for GnRH, 0.3–60 mlU/mL for LH, 0.17–10 mlU/mL for FSH, and 0.156–10 ng/mL for kisspeptin 1.

### Real-time qPCR

Total RNA was extracted from hypothalamic tissues using TRIzol reagent (Wuhan Servicebio Technology CO., LTD, China) and quantified with a NanoDrop 2000 spectrophotometer (Thermo Fisher Scientific, USA). The extracted RNA was reverse-transcribed into first-strand cDNA using SweScript All-in-One RT SuperMix (Wuhan Servicebio Technology CO., LTD, China). Quantitative PCR assays were performed on a real-time PCR system (Bio-Rad, USA). The PCR system consisted of 10 μL SYBR Green master mix, 1 μL 10 μM primer mix, 7 μL ddH2O, and 2 μL cDNA. *Gapdh* was used as the endogenous control, and mRNA expression is presented as 2^−ΔΔCT^. Primer sequences are provided in Table 1.

**Table 1:** Primer sequences used for RT-PCR.

Targeted genes	Primer sequence (5′→3′)
*Kiss1*	F: TCTCGCTGGCTTCTTGGC
	R: GGG​TTC​AGG​GTT​CAC​CAC​A
*Tac3*	F: TAG​CGT​GGG​ACC​GAA​GGA​GA
	R: TCA​ACC​ACA​TCA​GCG​GGA​GT
*Pdyn*	F: CGGAGGAGTGGGAGACAT
	R: GAC​GCT​GGT​AAG​GAG​TTG​G
*Gapdh*	F: CTG​GAG​AAA​CCT​GCC​AAG​TAT​G
	R: GGT​GGA​AGA​ATG​GGA​GTT​GCT

### Immunohistochemistry

Coronal sections, 3 μm thick, containing the ARC were prepared from the fixed brain tissue. The sections were sequentially deparaffinized, hydrated, and subjected to antigen retrieval, followed by incubation in 3% hydrogen peroxide solution (Sinopharm Chemical Reagent Co., Ltd, China) for 25 min to block endogenous peroxidase activity. After washing, the slides were treated with a serum-blocking solution (Servicebio, China) and incubated overnight at 4°C with primary antibodies: KISS1 antibody (1:20, Affinity-DF7133, China), rabbit anti-neurokinin B (NKB) polyclonal antibody (1:200, Beijing Biosynthesis Biotechnology Co., Ltd-bs-0070R, China), and rabbit anti-PDYN polyclonal antibody (1:100, Absin-abs116888, China). The slides were then washed and incubated with species-specific secondary antibodies (Servicebio, China) for 50 min at room temperature. Following three rinses in phosphate-buffered saline (PBS) (Servicebio, China), the sections were air-dried slightly, and freshly prepared DAB (3,3′-diaminobenzidine) solution was applied dropwise. The color development process was monitored under a microscope, and staining was terminated by rinsing with tap water. After counterstaining the nuclei with hematoxylin, the sections were dehydrated and mounted. Images of the ARC region were captured using CaseViewer software 2.0 (3DHISTECH Ltd, Hungary), and the average optical density of the immunohistochemical staining was measured using ImageJ image processing software (NIH, USA).

### Statistical analysis

Statistical analyses were performed using SPSS 23.0 (IBM Corp., USA), GraphPad Prism 9.0 (GraphPad Software, USA), and R software 4.3.3. The timing of preputial separation between the control and PNA groups was compared using the log-rank test. Differences in serum hormone levels, mRNA expression, and immunohistochemical indices at puberty onset between the two groups were analyzed using Student’s *t*-test. Hierarchical multiple regression, adjusted for puberty onset timing, was applied to assess the effects of prenatal testosterone intervention on serum hormone levels and mRNA expression in male offspring. Mixed linear models were employed to analyze differences in immunohistochemical indices between the control and PNA groups. Differences were considered statistically significant when *P* < 0.05.

## Results

### Effect of PNA on the onset of puberty in male offspring

**Figure 1:**
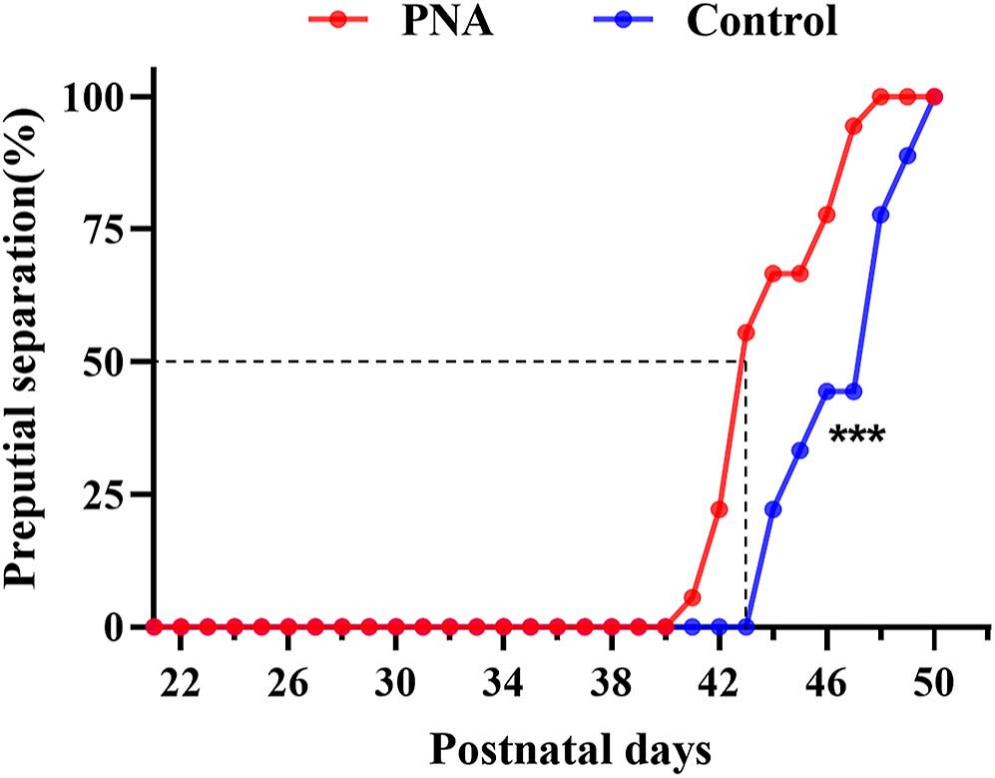
Cumulative percentage of male rats with preputial separation in control and PNA groups. Statistical significance was determined by the log-rank test. ***P < 0.001.

As shown in Fig. 1, the onset of puberty in male offspring from the PNA group occurred significantly earlier than in the control group (*P* < 0.001). The first instance of preputial separation in the PNA group was observed on PND41, compared to PND44 in the control group. In addition, over 50% of male rats in the PNA group had undergone preputial separation by PND42, whereas none of the male rats in the control group had exhibited this marker of puberty onset.

### Transcriptomic analysis of the hypothalamus in male offspring at PND21

**Figure 2:**
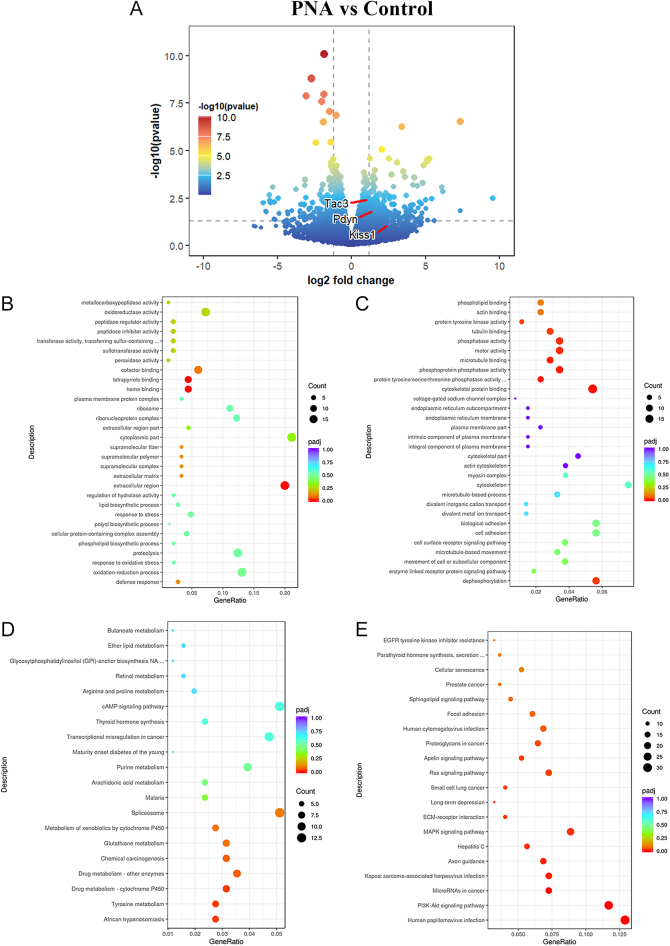
Transcriptome differences in the hypothalamus of PND21 male offspring between PNA and control groups. (A) Volcano plot of all detected genes. (B and C) GO enrichment analysis of up- and down-regulated genes. (D and E) KEGG pathway analysis of up- and down-regulated genes.

In the comparison of PND21 male offspring between the PNA and control groups, 1,315 genes were differentially expressed (*P* < 0.05, |log2 fold change| >0), including 648 up-regulated and 667 down-regulated genes. In the hypothalamus of PND21 male offspring from the PNA group, the expression of *Tac3* and *Pdyn* was significantly higher compared to controls (*P* < 0.01 for *Tac3*, and *P* < 0.05 for *Pdyn*), while changes in *Kiss1* expression were not significant (Fig. 2A, Supplementary Tables 1 and 2 (see section on Supplementary materials given at the end of the article)). The expressions of *Agrp* and *Pomc* genes, which are associated with sexual maturation, were also significantly increased (*P* < 0.05) (Supplementary Table 1).

GO enrichment analysis showed that up-regulated genes were primarily enriched in the molecular function categories of ‘heme binding’ and ‘tetrapyrrole binding’. In the cellular component category, DEGs were mainly located in the extracellular region (Fig. 2B). Conversely, down-regulated genes were enriched in the biological process of ‘dephosphorylation’ and were involved in molecular functions such as ‘cytoskeletal protein binding’, ‘protein tyrosine/serine/threonine phosphatase activity’, ‘phosphoprotein phosphatase activity’ and ‘microtubule binding’ (Fig. 2C).

KEGG pathway analysis revealed that up-regulated genes were enriched in pathways related to ‘African trypanosomiasis’, ‘tyrosine metabolism’, and ‘drug metabolism-cytochrome P450’ (Fig. 2D). Down-regulated genes were mainly enriched in pathways associated with ‘Human papillomavirus infection’, ‘PI3K–Akt signaling pathway’, ‘microRNAs in cancer’, and ‘Kaposi sarcoma-associated herpesvirus infection’ (Fig. 2E).

Notably, the differential expression of *Tac3* and *Pdyn* was enriched in the biological processes of the ‘G-protein-coupled receptor signaling pathway’ and the ‘neuroactive ligand–receptor interaction’ pathway. Although these enrichments were not statistically significant (*P* > 0.05), they may provide valuable insights into the potential mechanisms underlying the biological significance of this study.

### Effect of PNA on serum hormone levels in male offspring at the onset of puberty

**Figure 3:**
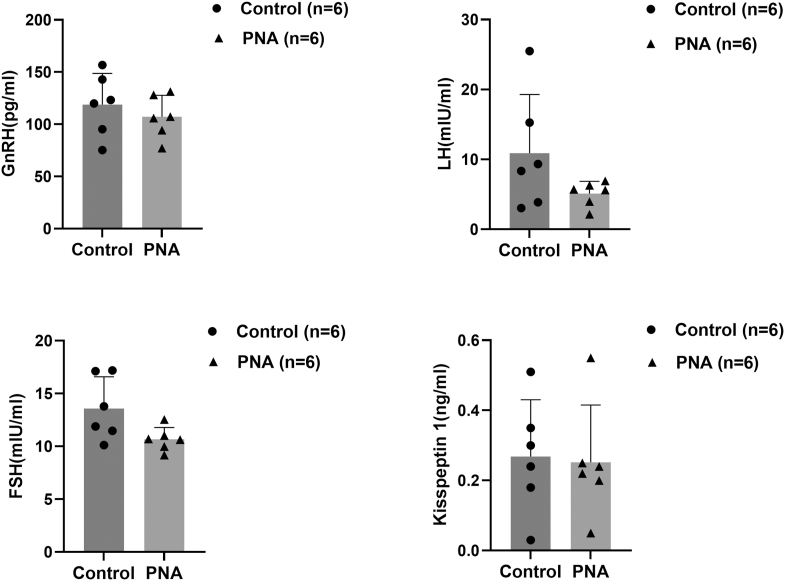
Effect of PNA on serum levels of GnRH, LH, FSH, and kisspeptin 1 in male offspring at the onset of puberty. No significant differences were observed between groups (*P* > 0.05).

As shown in Fig. 3, at the onset of puberty, the differences in serum levels of GnRH, LH, FSH, and kisspeptin 1 between male offspring in the PNA group and the control group were not statistically significant (*P* > 0.05). The impact of PNA on serum hormone levels in male offspring was further analyzed using hierarchical multiple regression, adjusting for the time of puberty onset. The results, presented in Table 2, indicate a positive correlation between the time of puberty onset and serum kisspeptin 1 levels (*P* < 0.05). However, the remaining models did not reach statistical significance (*P* > 0.05).

**Table 2:** Hierarchical multiple regression analysis of serum hormone levels in male offspring at the onset of puberty.

	GnRH (pg/mL)			LH (mlU/mL)			FSH (mlU/mL)			Kisspeptin 1 (ng/mL)		
	*B*	SE	*P*	*B*	SE	*P*	*B*	SE	*P*	*B*	SE	*P*
Timing of onset of puberty	1.195	3.353	0.730	0.755	0.757	0.345	0.163	0.292	0.091	0.041*	0.016	0.033
Groups	−10.541	15.755	0.520	−5.156	3.557	0.181	−2.786	1.374	0.073	0.018	0.077	0.823
*R^2^*	0.070			0.293			0.356			0.414		
*ΔR^2^*	0.046			0.165			0.294			0.003		
*F*	0.340			1.863			2.488			3.184		

Groups: 0, control; 1, PNA. SE*, *standard error.

* *P* < 0.05.

### Effect of PNA on hypothalamic mRNA expression in male offspring at the onset of puberty

**Figure 4:**
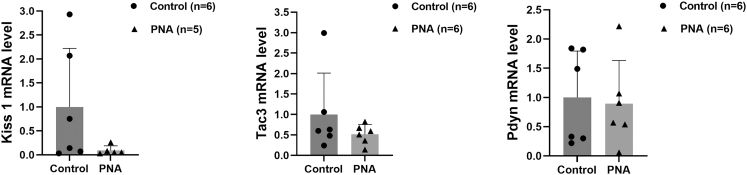
Effect of PNA on hypothalamic mRNA expression in male offspring at the onset of puberty. No significant differences were observed between groups (*P* > 0.05).

Regarding hypothalamic mRNA expression, an outlier was identified in the hypothalamic *Kiss1* mRNA levels of male offspring in the PNA group. This outlier was excluded, and the *Kiss1* mRNA data from five male offspring in the PNA group were ultimately used for statistical analysis. As shown in Fig. 4, the differences in hypothalamic *Kiss1*, *Tac3*, and *Pdyn* mRNA levels between the PNA group and the control group were not statistically significant (*P* > 0.05). The effect of PNA on hypothalamic gene expression in male offspring was further analyzed using hierarchical multiple regression, adjusted for the time of puberty onset, as presented in Table 3. However, none of the models reached statistical significance (*P* > 0.05).

**Table 3:** Hierarchical multiple regression analysis of hypothalamic mRNA expression in male offspring at the onset of puberty.

	*Kiss1*			*Tac3*			*Pdyn*		
	*B*	SE	*P*	*B*	SE	*P*	*B*	SE	*P*
Timing of onset of puberty	−0.142	0.114	0.249	0.104	0.090	0.276	−0.088	0.097	0.389
Groups	−0.971	0.540	0.110	−0.398	0.424	0.372	−0.178	0.454	0.704
*R^2^*	0.354			0.230			0.089		
*ΔR^2^*	0.261			0.075			0.016		
*F*	2.191			1.348			0.438		

Groups: 0, control; 1, PNA. SE*, *standard error.

### Effect of PNA on protein expression in the ARC of male offspring at the onset of puberty

**Figure 5:**
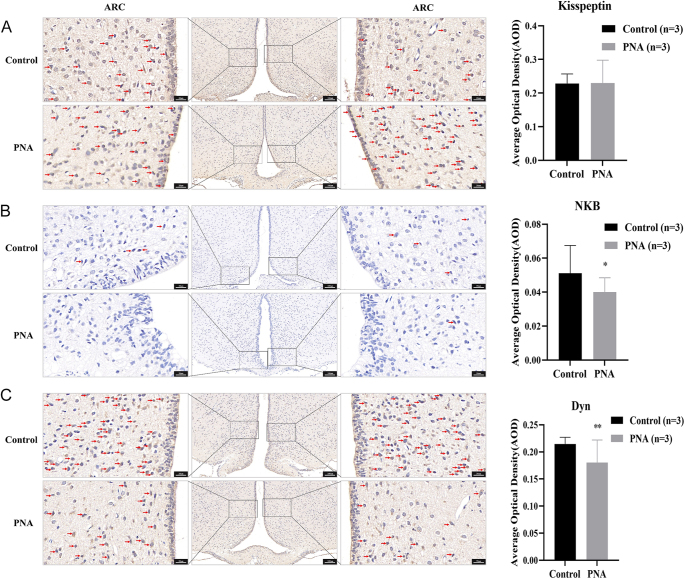
Expression of kisspeptin, NKB, and Dyn in the ARC of male offspring at the onset of puberty. *P < 0.05; **P < 0.01.

Figure 5 illustrates the expression levels of kisspeptin, NKB, and Dyn in the ARC of male offspring from both the control and PNA groups. The results were analyzed using mixed linear models, with group as a fixed effect variable and puberty onset time as a random effect variable. The findings, summarized in Table 4, show a positive correlation between puberty onset time and kisspeptin expression. Adjusted for the time of puberty onset, the effect of PNA on the expression of kisspeptin and Dyn in the ARC was significant (*P* < 0.01). Male offspring in the PNA group may have exhibited significantly higher kisspeptin expression in the ARC compared to the control group (*P* < 0.01). In contrast, their expression of Dyn in the ARC was significantly lower than that of the control group (*P* < 0.01).

**Table 4:** Mixed linear model analysis of kisspeptin, NKB, and Dyn expression in the ARC of male offspring at the onset of puberty.

Target protein	Variable	Estimated coefficient	SE	*t*	*P*
Kisspeptin	Intercept	−0.714	0.165	−4.33	<0.001
	Timing of onset of puberty	0.020	0.003	5.73	<0.001
	Groups	0.054	0.016	3.48	0.001
NKB	Intercept	0.010	0.059	0.17	0.866
	Timing of onset of puberty	0.001	0.001	0.69	0.494
	Groups	−0.009	0.006	−1.67	0.105
Dyn	Intercept	0.380	0.133	2.85	0.007
	Timing of onset of puberty	−0.003	0.003	−1.24	0.223
	Groups	−0.044	0.013	−3.50	0.001

Groups: 0, control; 1, PNA. SE*, *standard error*.*

## Discussion

Exposure to excessive androgens during early life has been linked to various chronic and malignant diseases (33, 34, 35). In this study, we investigated the effect of androgen excess during late pregnancy on puberty onset in male offspring using a hyperandrogenic pregnant rat model. Our findings indicate that *in utero* exposure to elevated androgen levels led to an earlier onset of puberty in male offspring. Moreover, male offspring in the PNA group exhibited upregulation of *Tac3*, a key gene involved in HPG axis activation, during the juvenile stage. Further analysis of the effects of PNA on male offspring at puberty onset revealed significant changes in the ARC, specifically in the expression of kisspeptin and dynorphin, proteins closely linked to puberty onset. These results suggest a potential mechanism by which hyperandrogenic exposure during late pregnancy may influence the timing of puberty onset by altering the secretion of KNDy neurons in the offspring.

Puberty onset occurs when the pulsatile secretion of gonadotropin-releasing hormone (GnRH) is reactivated, following suppression during childhood (12). Premature pulsatile GnRH secretion from the hypothalamus may result from an increase in excitatory or a decrease in inhibitory signaling within the HPG axis during childhood, leading to premature reactivation of the axis and early puberty onset. The hypothalamus regulates GnRH pulses through a complex network of neurons and glia, among which KNDy neurons are of particular interest (36). Mutations in the *Kiss1* gene have been linked to hypogonadotropic hypogonadism and pubertal developmental disorders in humans (37). Similarly, mutations in *Tac3* or *Tacr3*, which encode NKB and its receptor, have been associated with this phenotype (38). Our results show that male offspring in the PNA group exhibited significantly higher expression of *Tac3* and *Pdyn* in the hypothalamus during the juvenile stage compared to controls. Although *Kiss1* expression was elevated, the difference was not statistically significant. These findings suggest that exposure to hyperandrogenism during late pregnancy may affect the onset of puberty in male offspring by altering KNDy neurons, influencing the timing of puberty. Specifically, PNA exposure led to the upregulation of *Tac3* at PND21, a key gene in activating the HPG axis during the juvenile stage. However, this activation was mitigated by elevated *Pdyn*, resulting in a less pronounced increase in *Kiss1* expression. Consequently, the HPG axis may not have been fully reactivated during this period. Consistent with our findings, a previous study on hypothalamic mRNA levels in PNA-induced female offspring at 4 weeks of age also reported no significant difference in *Kiss1* mRNA expression between the PNA and control groups, but a significant increase in hypothalamic *Tac3* mRNA expression in PNA-treated females (39). In addition, we found that the DEGs *Tac3* and *Pdyn* were enriched in biological processes related to the ‘G-protein-coupled receptor signaling pathway’ and the ‘neuroactive ligand–receptor interaction’ pathway. Although these enrichment results were not statistically significant, they suggest a potential pathway through which PNA may influence pubertal development. G protein-coupled receptor 54 (GPR54), a key receptor for kisspeptin, is a major regulator of GnRH and gonadotropin secretion (40), and plays a crucial role in the regulation of pubertal development (41). Notably, transcriptomic results from this study revealed that the expression of *Agrp* and *Pomc* genes associated with sexual maturation was significantly elevated. Studies indicate that *Pomc*-derived α-MSH can activate MC4R receptors within KNDy neurons via the melanocortin signaling pathway, promoting the release of NKB and kisspeptin to drive GnRH pulse secretion (42). Conversely, *Agrp* neurons may inhibit GnRH pulses by suppressing MC4R signaling or enhancing dynorphin release within KNDy neurons (43). This is consistent with our core findings, suggesting that *Pomc* and *Agrp* may act as upstream regulators of KNDy neurons to participate in the regulatory process of pubertal timing.

Our results showed that androgen exposure during late pregnancy significantly advanced the onset of puberty in male offspring. To further elucidate the mechanism by which PNA causes the early onset of puberty in male offspring, we aimed to validate and extend the primary findings from the RNA-seq analysis conducted during the juvenile stage in male offspring at the onset of puberty. ELISA and qPCR analyses demonstrated no statistically significant differences in serum concentrations of GnRH, LH, FSH, and kisspeptin 1, or hypothalamic mRNA expression levels of *Kiss1*, *Tac3*, and *Pdyn* between the PNA group and control (*P* > 0.05). These findings suggest that the effects of prenatal androgen exposure on circulating hormone levels may diminish progressively during development. This observation, combined with transcriptomic evidence from hypothalamic tissue at PND21 (*Tac3* upregulation), indicates that critical developmental programming was likely established during earlier stages (PND21). Furthermore, prenatal androgen exposure may lower the intrinsic stimulation threshold required for HPG axis activation in male offspring. Given that post-transcriptional modifications or variations in the regulation and modification of the translational process can result in discrepancies between protein and mRNA expression, we used immunohistochemistry to localize the ARC in male offspring at the onset of puberty and detect target protein levels in this region. Our results demonstrated a significant increase in kisspeptin expression and a significant decrease in dynorphin expression in the ARC of the PNA group compared to controls, after adjusting for the time of puberty onset. KNDy neurons located in the ARC regulate NKB and dynorphin through synaptic connections to fine-tune the release of kisspeptin and GnRH (44). NKB stimulates kisspeptin release by binding to NK3R on KNDy neurons, with kisspeptin subsequently binding to GPR54 to activate GnRH neurons. The release of dynorphin by KNDy neurons terminates the GnRH pulse. Previous studies have demonstrated that bisphenol A exposure promotes early puberty onset by increasing *Kiss1* expression in the ARC of juvenile female rats (45). In addition, chronic central administration of *Kiss1* peptide in immature female rats advanced the timing of vaginal opening and significantly increased LH levels (46). Makorin ring finger protein 3 (MKRN3) has also been shown to play an important role in puberty onset, and deletion of MKRN3 in female mice led to earlier puberty and was associated with increased NKB levels in the ARC at PND25 (47). Administration of insulin-like growth factor 1 (IGF-1) in female rats also resulted in earlier puberty onset, accompanied by increased NKB expression and decreased dynorphin expression (48). These findings suggest that PNA may reduce dynorphin expression in the ARC of male offspring, diminishing its inhibitory effect. This could lead to increased kisspeptin secretion in the ARC, ultimately resulting in premature activation of GnRH neurons and earlier onset of puberty. However, the differences in kisspeptin, serum GnRH, LH, and FSH levels between the PNA group at puberty onset and controls were not statistically significant (*P* > 0.05). This may be attributed to the pulsatile nature of GnRH release, which causes fluctuations in serum LH and FSH concentrations.

The secular trend toward earlier puberty onset has emerged as a global public health challenge (49). Our findings suggest that prenatal hyperandrogenic exposure may act as a critical contributing factor to this phenomenon. Specifically, KNDy neurons in the ARC play a pivotal role in mediating the accelerated puberty onset observed in male offspring exposed to elevated testosterone levels during gestation. Targeted interventions modulating the KNDy signaling pathway hold promise for disrupting this adverse developmental trajectory.

## Conclusion

Our study demonstrates that KNDy neurons in the ARC play a crucial role in the onset of puberty in male offspring exposed to elevated testosterone levels during late pregnancy. Specifically, prenatal androgen exposure may lead to a reduction in Dyn–KOR signaling in the ARC, which enhances kisspeptin–GPR54 signaling. This, in turn, results in the premature activation of GnRH neurons and the early onset of puberty.

## Supplementary materials



## Declaration of interest

The authors declare that this study was conducted in the absence of any commercial or financial relationships that could be construed as potential conflicts of interest.

## Funding

This work was supported by the General Program of Natural Incubation of Bengbu Medical University (2022byfy001); the 512 Talent Training Program of Bengbu Medical University (by51201204); the Overseas Visiting and Training Programs for Outstanding, Young Core Talents in Universities (gxgwfx2020042); Anhui Provincial University Scientific Research Project (2023AH040285); and the Longhu Talent Project of Bengbu Medical University (LH250203002).

## Author contribution statement

Runfei Ge and Yun Zhang contributed to formal analysis, visualization, original draft writing, and investigation. Yongting Yuan, Tingting Li, and GuiYu Qiu were responsible for data curation, investigation, methodology, and validation. Shuaijun Guo contributed to supervision, validation, and project administration. Lianguo Fu was responsible for conceptualization, data curation, funding acquisition, methodology, as well as writing, review, and editing.

## Data availability

The RNA-seq data generated in this study have been deposited in the NCBI Gene Expression Omnibus (GEO) under accession number PRJNA1263749. The data used to support the findings of this study are available from the authors upon reasonable request.

## Ethics statement

All experimental procedures were approved by the Ethics Committee of Bengbu Medical University (Approval No. (2018) 032).
