# Supplementary material for: Role of KNDy neurons in puberty onset in male offspring following prenatal androgen exposure
Source: Endocr Connect. 2025 Jul 17;14(7):e250209. doi: 10.1530/EC-25-0209 (PMC12278364; doi:10.1530/EC-25-0209)
Supplement: Supplementary file 1 [file supplementary_materials.pdf]

Supplementary Table 1 Top 50 significantly upregulated and downregulated genes in PNA vs. Control male offspring at PND21

Gene name	log2FoldChange	Regulation	*P*	padj	chromosomal locations
Cga	9.541	Up	0.003	0.334	chr5:50381244-50393367
Np4	7.356	Up	<0.001	0.001	chr16:75414786-75481115
Tshb	6.190	Up	0.001	0.243	chr2:205207799-205212681
NMS	6.118	Up	0.001	0.182	chr9:45605552-45616912
RatNP-3b	5.227	Up	<0.001	0.027	chr16:75497832-75530517
Defa5	5.138	Up	<0.001	0.030	chr16:75338052-75340360
Camp	5.094	Up	<0.001	0.027	chr8:117930546-117932518
Pitx1	5.036	Up	0.011	0.489	chr17:8878270-8884423
Fndc3c1	5.008	Up	0.004	0.362	chrX:77507814-77559348
Ces1d	4.863	Up	<0.001	0.040	chr19:15033108-15239821
Pomc	4.788	Up	0.001	0.152	chr6:28382962-28388967
Mpo	4.438	Up	0.001	0.180	chr10:75087892-75098260
LOC24906	4.232	Up	<0.001	0.105	chr10:43871613-43880055
AABR07051308.1	4.173	Up	0.001	0.175	chr3:4730990-4772154
LOC100911750	4.153	Up	0.007	0.440	chr1:220416018-220418240
Fcnb	4.000	Up	0.012	0.495	chr3:6617751-6626284
Six2	3.994	Up	0.008	0.451	chr6:8952572-8956276
AC103129.3	3.708	Up	0.002	0.301	chr7:140154490-140172448
Ngp	3.608	Up	<0.001	0.097	chr8:118662335-118665699
AABR07044962.1	3.591	Up	0.009	0.478	chr20:28365988-283

					75791
S100a8	3.475	Up	<0.001	0.067	chr2:190073815-190074354
Yif1b	3.404	Up	<0.001	0.001	chr1:87180624-87191185
S100a9	3.079	Up	<0.001	0.032	chr2:190097554-190100276
Agrp	2.877	Up	0.003	0.327	chr19:37526419-37528011
Pomp	2.870	Up	0.007	0.436	chr12:8746855-8759599
Nmu	2.835	Up	0.001	0.180	chr14:34354503-34381828
AC096370.2	2.723	Up	0.007	0.439	chr1:259641676-259674425
Gal	2.708	Up	<0.001	0.087	chr1:218652917-218657925
Vdr	2.697	Up	0.003	0.327	chr7:139342063-139394166
Mmp8	2.595	Up	0.003	0.334	chr8:5768811-5778702
-	2.559	Up	0.003	0.319	chrX:156761489-156771186
AABR07024041.2	2.490	Up	<0.001	0.027	chrAABR07024041.1:23532-31048
LOC287167	2.323	Up	0.002	0.256	chr10:15577249-15577977
LOC100361018	2.306	Up	0.003	0.320	chr20:29951637-29952023
LOC100912571	2.261	Up	0.011	0.492	chr11:82886878-82893845
Clec10a	2.212	Up	0.005	0.386	chr10:56764927-56769457
Adh6	2.195	Up	0.009	0.478	chr2:243550627-243687857
Sytl4	2.118	Up	0.002	0.312	chr2:243550627-243687857
Tnnt2	2.053	Up	0.007	0.443	chr13:52662996-52680990
Capg	2.039	Up	<0.001	0.011	chr4:100407658-100419446
Cldn3	2.034	Up	0.011	0.492	chr12:21831342-21832813
LOC100911576	2.019	Up	0.011	0.495	chr12:21831342-218

					32813
AC127013.1	2.018	Up	0.012	0.495	chr4:155088317-155092827
Hba1	1.907	Up	0.002	0.279	chr10:15602794-15603649
Uchl3	1.899	Up	0.011	0.492	chr3:171092946-171134655
Ptgds	1.853	Up	0.002	0.279	chr3:2686123-2689084
Fxyd3	1.794	Up	0.006	0.428	chr1:89502562-89509343
Pdyn	1.782	Up	0.011	0.492	chr3:122194329-122206671
S100a6	1.777	Up	0.005	0.400	chr2:190007216-190008511
Pdlim3	1.739	Up	0.005	0.393	chr16:49543142-49574390
-	-5.983	Down	0.006	0.409	chr2:194479757-194481296
-	-5.342	Down	0.001	0.180	chr11:34535992-34539032
Mroh4	-4.971	Down	0.003	0.334	chr7:115842960-115874418
AABR07026032.1	-3.744	Down	0.001	0.213	chr16:60008769-60018283
Rn60_10_0701.2	-3.424	Down	0.007	0.436	chr10:70398995-70400317
Tmco5a	-3.208	Down	0.007	0.446	chr3:108544931-108559476
Rlim	-3.205	Down	0.007	0.439	chr3:108544931-108559476
Fam131a	-3.153	Down	<0.001	0.114	chr11:82875627-82884660
LOC100911363	-3.152	Down	0.001	0.175	chr1:91058529-91063928
AABR07034750.1	-3.087	Down	0.003	0.334	chr11:87345865-87360080
Hnrnpab	-3.066	Down	<0.001	<0.001	chr10:34955716-34961608
AABR07061980.1	-2.884	Down	0.011	0.492	chr4:155752447-155763500
-	-2.790	Down	0.010	0.484	chr5:91126570-91130634
LOC100909750	-2.725	Down	<0.001	<0.001	chr3:9410661-94481

					67
AABR07049880.1	-2.615	Down	0.006	0.412	chr5:142303636-142330854
-	-2.399	Down	<0.001	0.006	chr4:96528368-96561724
Fam64a	-2.363	Down	0.011	0.489	chr10:58613674-58618514
AABR07019083.2	-2.211	Down	0.011	0.489	chr15:87390486-87390569
Frem3	-2.160	Down	0.002	0.288	chr19:30973329-31031817
-	-2.096	Down	0.002	0.301	chr4:28343132-28353521
Oprd1	-2.054	Down	0.011	0.494	chr5:150288126-150323063
Ewsr1	-2.045	Down	0.001	0.213	chr14:85322296-85350948
-	-2.037	Down	<0.001	<0.001	chr14:46640295-46645870
Kcnj4	-1.984	Down	0.004	0.359	chr7:120717375-120744602
AABR07015078.1	-1.948	Down	0.001	0.172	chr14:46649971-46650381
AABR07015057.1	-1.911	Down	<0.001	0.001	chr14:46523594-46529375
Fam111a	-1.885	Down	0.005	0.394	chr1:229003961-229019527
-	-1.864	Down	<0.001	<0.001	chr14:46639935-46644294
-	-1.848	Down	0.005	0.391	chr1:24984625-24996970
-	-1.833	Down	<0.001	<0.001	chr1:11907711-11909872
Clca1	-1.750	Down	0.003	0.327	chr2:250897969-250923744
-	-1.715	Down	0.003	0.323	chr7:101935064-101958457
Dmrta2	-1.686	Down	0.006	0.426	chr5:129756149-129758986
-	-1.677	Down	0.012	0.495	chr17:23245809-23549727
Col19a1	-1.660	Down	0.010	0.489	chr9:30515089-30844199
LOC100912253	-1.624	Down	0.008	0.451	chr17:15405995-154

					29051
AABR07003030.2	-1.623	Down	0.008	0.451	chr1:94027897-94152068
M6pr	-1.611	Down	0.003	0.322	chrX:88312675-88321834
LOC100911918	-1.601	Down	0.002	0.296	chr18:14756684-14964991
Casr	-1.583	Down	0.003	0.327	chr11:67188630-67258771
Grm2	-1.528	Down	0.011	0.492	chr8:115344999-115358046
AABR07053406.1	-1.520	Down	0.004	0.363	chr3:104816987-105086436
AABR07015081.2	-1.513	Down	<0.001	<0.001	chr14:46653040-46657975
Pcdh12	-1.508	Down	<0.001	0.105	chr18:31414250-31430973
AY172581.14	-1.500	Down	0.008	0.451	chrMT:2665-2739
Cd34	-1.496	Down	<0.001	0.118	chr13:113691932-113711647
Helz2	-1.478	Down	0.007	0.436	chr3:176731769-176744377
-	-1.474	Down	0.009	0.474	chr19:26267345-26563276
Col20a1	-1.470	Down	0.003	0.327	chr3:176494768-176526018
Cecr6	-1.447	Down	0.006	0.433	chr4:153026411-153028129

Padj: Adjusted p-value (for multiple hypothesis testing). Top 50 upregulated Genes: Genes were primarily sorted by log2FoldChange in descending order. Selection prioritized genes meeting Padj < 0.05. If fewer than 50 genes met this threshold, genes with 0.05 ≤ Padj < 0.5 were added, maintaining the highest log2FoldChange order; Top 50 downregulated Genes: Genes were primarily sorted by absolute log2FoldChange in descending order. Selection prioritized genes meeting Padj < 0.05. If fewer than 50 genes met this threshold, genes with 0.05 ≤ Padj < 0.5 were added, maintaining the largest absolute log2FoldChange order.

Supplementary Table 2 Expression analysis of Tac3 and Kiss1 in PNA vs. Control male offspring at PND21

Gene name	Log2 fold change	Regulation	*P*	*padj*	chromosomal locations
Tac3	1.517	Up	0.003	0.342	chr7:71023976-71030582
Kiss1	2.745	NS	0.057	0.668	chr13:50529510-50535389

Padj: Adjusted p-value (for multiple hypothesis testing). $P < 0.05$ considered nominally significant; NS: Not statistically significant ($P > 0.05$).
